# Comparative analysis of powdery mildew resistant and susceptible cultivated cucumber (*Cucumis sativus* L.) varieties to reveal the metabolic responses to *Sphaerotheca fuliginea* infection

**DOI:** 10.1186/s12870-020-02797-3

**Published:** 2021-01-07

**Authors:** Peng Zhang, Yuqiang Zhu, Shengjun Zhou

**Affiliations:** Institute of Vegetable, Zhejiang Academy of Agriculture Sciences, Hangzhou, China

**Keywords:** Fatty acid, Flavonoid, Hormone, Metabolism, Powdery mildew, Transcription factor

## Abstract

**Background:**

Cucumber (*Cucumis sativus* L.) is a widely planted vegetable crop that suffers from various pathogen infections. Powdery mildew (PM) is typical disease caused by *Sphaerotheca fuliginea* infection and destroys the production of cucumber. However, the metabolic responses to *S. fuliginea* infection are largely unknown.

**Results:**

In our study, a PM resistant variety ‘BK2’ and a susceptible variety ‘H136’ were used to screen differentially accumulated metabolites (DAMs) and differentially expressed genes (DEGs) under *S. fuliginea* infection. Most of DEGs and DAMs were enriched in several primary and secondary metabolic pathways, including flavonoid, hormone, fatty acid and diterpenoid metabolisms. Our data showed that many flavonoid-related metabolites were significantly accumulated in BK2 rather than H136, suggesting an essential role of flavonoids in formation of resistant quality. Changes in expression of *CYP73A*, *CYP81E1*, *CHS*, *F3H*, *HCT* and *F3’M* genes provided a probable explanation for the differential accumulation of flavonoid-related metabolites. Interestingly, more hormone-related DEGs were detected in BK2 compared to H136, suggesting a violent response of hormone signaling pathways in the PM-resistant variety. The number of fatty acid metabolism-related DAMs in H136 was larger than that in BK2, indicating an active fatty acid metabolism in the PM-susceptible variety.

**Conclusions:**

Many differentially expressed transcription factor genes were identified under *S. fuliginea* infection, providing some potential regulators for the improvement of PM resistance. PM resistance of cucumber was controlled by a complex network consisting of various hormonal and metabolic pathways.

## Background

Cucumber (*Cucumis sativus* L.) is an economically significant vegetable crop widely planted in many countries (http://faostats.fao.org). Although cucumber is important, its production is largely limited by various infective agents, including bacterial, viral, fungal and oomycete [1, 2]. Powdery mildew (PW) is typical disease that was caused by *Sphaerotheca fuliginea* infection and destroys the production of cucumber in many regions [3]. Recently, many works have been done to reveal the epidemiology, host specificity, and genome of *S. fuliginea* [4, 5]. Beginning with a conidial spore, PM fungus builds several germ tubes to invade the epidermal cells of host plants [6]. After breaking through the cell wall barrier, *S. fuliginea* develops a haustorium and absorbs nutrients from the host plants [6, 7].

The pathogen *S. fuliginea* has a broad host range, particularly in cucurbit crops, and its quick infection process makes PM disease difficult to be controlled in the field [8, 9]. Genetic breeding for PM-resistant cucumber lines is an effective approach to control this destructive disease [10]. In the past years, a number of PM-resistance-related genes and metabolites have been identified in various cucumber varieties [8, 11–13]. Two cucumber translationally controlled proteins, CsTCTP1 and CsTCTP2, were identified as negative regulators in the defense responses to *S. fuliginea* infection [14]. *CsPTI1* encoding a cytoplasmic kinase involved in defense responses to the fungal pathogen *S. fuliginea* [15]. Fine mapping of a major-effect QTL, Pm1.1, identifies two cysteine-rich receptor protein kinase encoding genes as cucumber PM resistance candidate genes [16]. *CsaMLO8* is identified as a functional susceptibility gene to PM disease, and its loss-of-function mutation results in hypocotyl resistance to PM in cucumber seedlings [17]. Several secondary metabolites have been reported to be involved in the resistance to PM. In cucumber, biosynthesis of flavonoid phytoalexins compounds plays a role in the rapid induction of PM disease resistance [18]. A flavonol aglycone rhamnetin was reported as a flavonol phytoalexin in cucumber [19]. Additionally, several phytohormones, such as auxin, abscisic acid (ABA), gibberellin (GA), cytokinin (CTK), and brassinolide (BR), showed quick responses to pathogen infections [20–22].

Technical advances in large-scale screening of genes and proteins have applied to investigate the responses of cucumber to *S. fuliginea* infection [23]. Recently, a comparative transcriptome profiling identified several candidate genes and provided valuable information for the fine mapping of Pm5.1, a well-known PM resistant segment [24]. Physcion and chrysophanol are two chemical compounds that trigger defense responses against PM in cucumbers [25]. Transcriptomic analysis revealed distinct resistant responses that were induced by physcion and chrysophanol to cucumber PM infection [26]. Whole-genome resequencing of cucumber have identified many candidate genes governing PM resistance [27].

In the past years, many PM-resistant cucumber varieties, including PI198088 line, European greenhouse type S06, inbred S1003, WI2757 and BK2, have been developed [8, 11–13, 28]. These resistant lines and varieties are valuable materials for screening of PM resistant genes [29]. However, the metabolic responses of cucumber plants to *S. fuliginea* infection are largely unknown. To reveal the distinct responses of PM-resistant and PM-susceptible cucumber varieties, two cultivated cucumber varieties were used for transcriptomic and metabolomic analyses. Our results might allow us to screen more potential genes and metabolites associated with the PM resistance.

## Results

### Overview of the transcriptome

RNA sequencing yields a total of 89.34 Gb valid sequence data, including 21.64 Gb from BK2C, 22.64 Gb from BK2T, 22.27 Gb from H136C, and 22.79 Gb from H136T (Additional file 1). Most of the clean reads were mapped onto the cucumber reference genome (NCBI: ASM407v2), including 83.56% unique mapped reads and 12.98% multiple mapped reads. Analysis of mapped region showed that 93.75% reads were mapped on exon regions, 4.27% reads were mapped on intron regions, and 1.99% reads were mapped on intergenic regions (Additional file 2). After searching the cucumber genome database, 24,317 cucumber genes with annotation were obtained (Additional file 3).

### Identification of DEGs in different comparsions

**Fig. 1:**
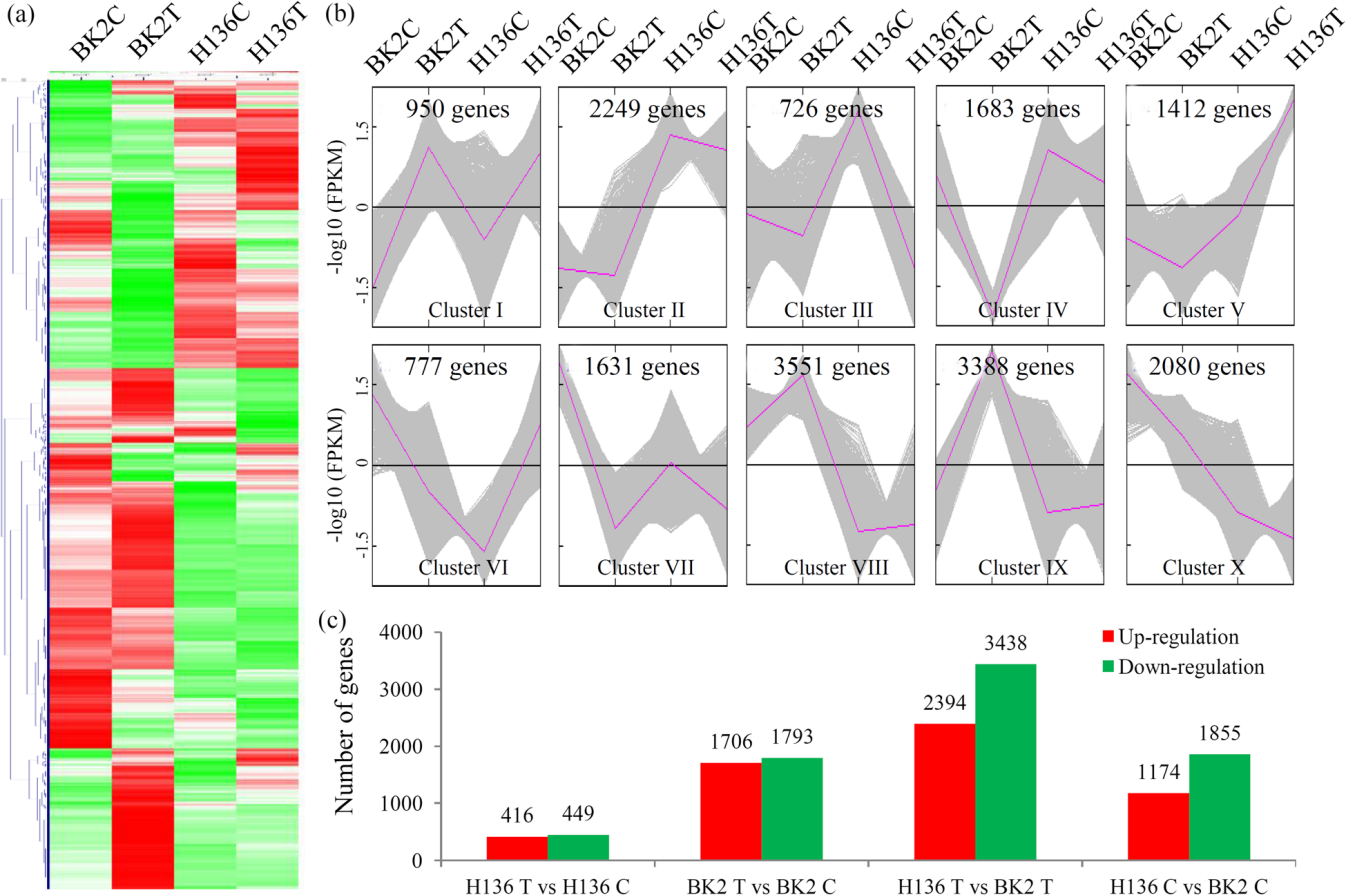
Analysis of the differentially expressed genes (DEGs) under *S. fuliginea* infection. **a** Heatmap for cluster analysis of the differentially expressed genes by K-mean method. Red indicated high expressed genes and green indicated low expressed genes. The heatmap scale ranges from − 2 to + 2 on a log2 scale. **b** MeV cluster analysis of the DEGs from the gene expression profiles. **c** The number of up- and down-regulated genes in different comparisons

An overview of the expression profiles of all identified genes is shown by a heatmap (Fig. 1a). All identified genes were grouped into 10 major clusters by K-means clustering method. Cluster I included the genes that were induced by *S. fuliginea* infection in both BK2 and H136; Clusters VII and X included the genes that were reduced by *S. fuliginea* infection in both BK2 and H136; Cluster VI included the genes that were down-regulated in BK2 and up-regulated in H136; and Cluster III included the genes that were up-regulated in BK2 and down-regulated in H136 (Fig. 1b). According to the criation of |log2foldchange| > 1 and *P* < 0.05, a large number of DEGs were identified in different comparisons, including 416 up- and 449 down-regulated genes in the H136T vs H136C comparison, 1706 up- and 1973 down-regulated genes in the BK2T vs H136C comparsion, 2394 up- and 3438 down-regulated in the H136T vs BK2T comparison, and 1174 up- and 1855 down-regulated in the H136C vs BK2C comparison (Fig. 1c).

### Overview of the metabolomes

To explore the metabolic variations under *S. fuliginea* infection, an untargeted metabolomic approach was applied, yielding 7000 annotated metabolites from 10,341 ion features (Additional file 4). Analysis of several quality parameters, such as total ion chromatograms (TICs) and width, retenion-time of *m/z* and coefficent of variation (CV), showed that the sample preparation reached the sampling standard and the MS data could be used for further analysis (Additional file 5). Furhtermore, analysis of the variation parameter principal component (PC) showed that PC1 and PC2 were 48.75 and 18.12%, suggesting a greater difference in H136 than BK2 under *S. fuliginea* infection (Additional file 5).

Based on the KEGG annotation, a large number of metabolites were grouped into at least one KEGG category (Additional file 6). The top five largest KEGG terms were ‘diterpenoid biosynthesis’ (92 metabolites), ‘2-oxocarboxylic acid metabolism’ (77 metabolites), ‘biosynthesis of amino acids’ (74 metabolites), ‘arachidonic acid metabolism’ (71 metabolites), and ‘sesquiterpenoid and triterpenoid biosynthesis’ (67 metabolites).

### Idnetification of the DAMs in different comparsions

**Fig. 2:**
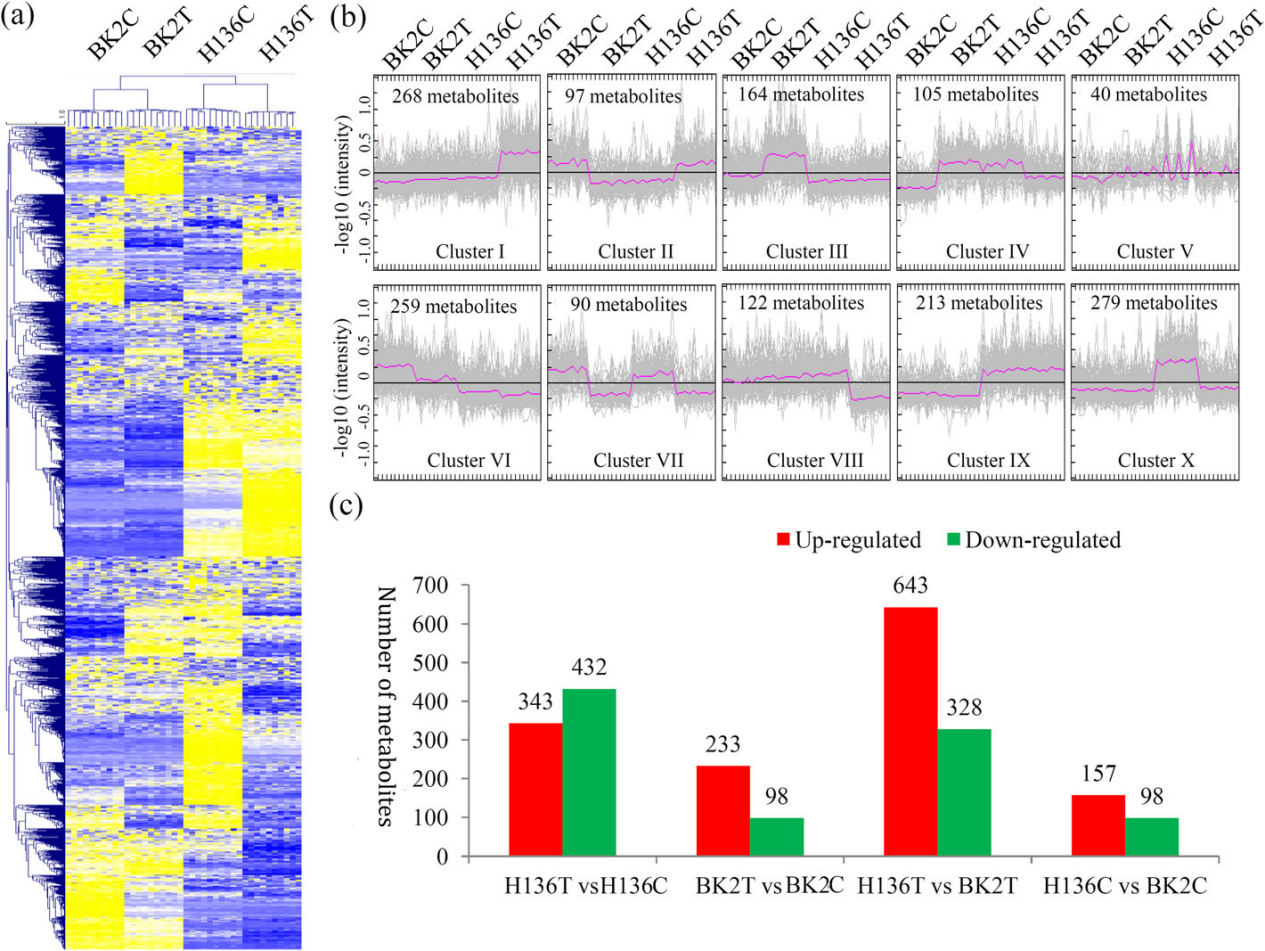
The variations in the abundance of metabolites from different samples. **a** A heatmap of the metabolites identified in the different cucumber metabolomes (*N* = 10). Yellow indicated high accumulated metabolites and blue indicated low accumulated metabolites. The heatmap scale ranges from − 2 to + 2 on a log2 scale. **b** MeV cluster analysis of the differentially accumulated metabolites from different samples. **c** The number of up- and down-regulated metabolites in different comparisons

After quality filtering, 1637 metabolites with KEGG annotation were used for fruther analysis. The metabolite profiling of two cucumber variaties under *S. fuliginea* infection is shown in Fig. 2a. All identified metabolites were clustered into 10 clusters to screen the DAMs. The metabolites only up-regulated in H136H were grouped into the Cluster I (268 metabolites); the metabolites only up-regulated in BK2 were classed into the Cluster III (164 metabolites); the metabolites only down-regulated in H136 were grouped into the Cluster VIII (122 metabolites) and X (279 metabolites); and the metabolites down-regulated in both of BK2 and H136 were the Cluster VII (90 metabolites) (Fig. 2b).

A number of DAMs were identified in different comparisons, including 343 up- and 432 down-regulated metabolites in the H136T vs H136C comparison, 233 up- and 98 down-regulated metabolites in the BK2T vs BK2C comparsion, 643 up- and 328 down-regulated metabolites in the H136T vs BK2T comparison, and 157 up- and 98 down-regulated metabolites in the H136C vs BK2C comparison (Fig. 2c).

### Variations in primary and secondary metabolic pathways under *S. fuliginea* infection

**Fig. 3:**
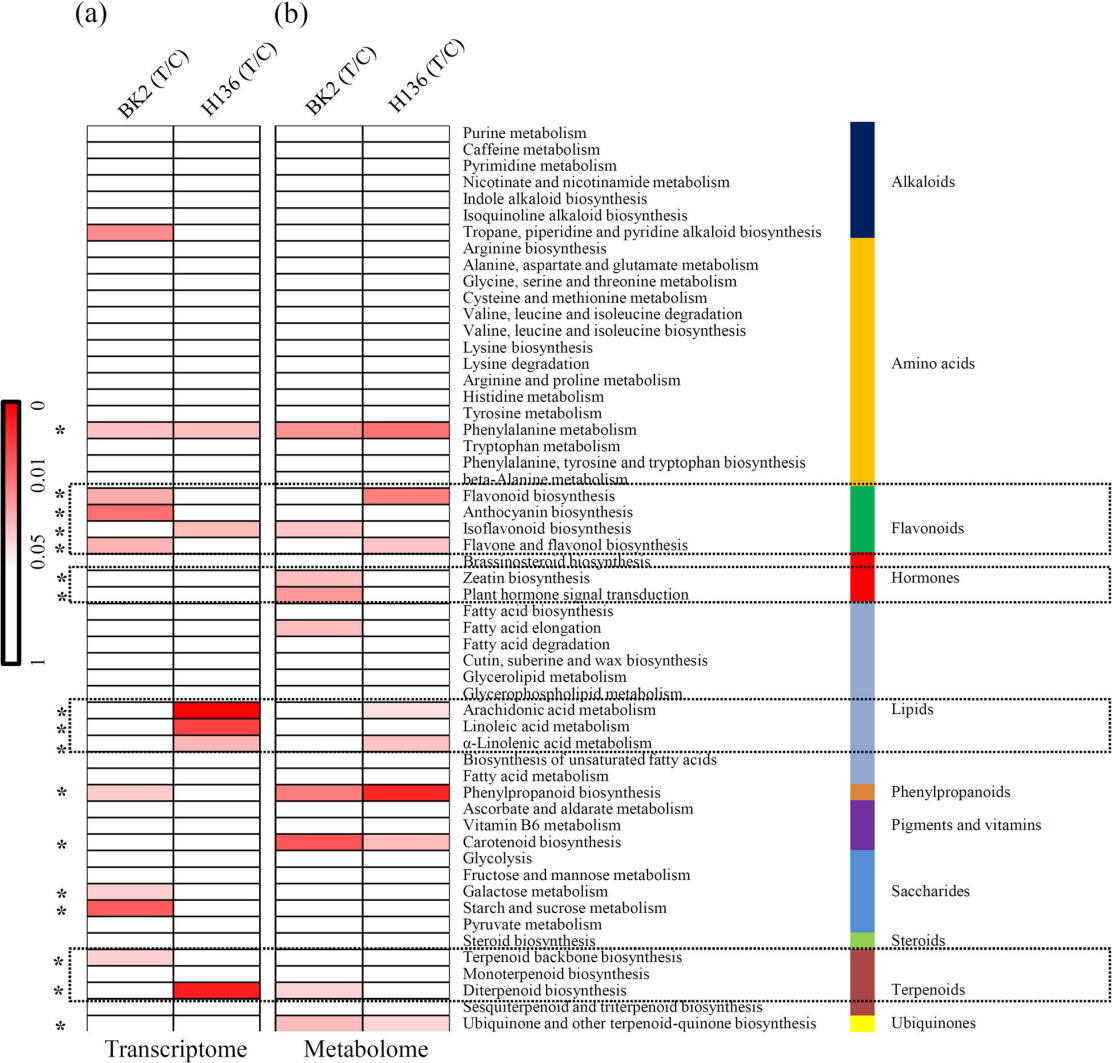
Comparative analysis of DEGs and DAMs of cucumber seedlings under the *S. fuliginea* infection. **a** KEGG enrichment analysis of the DEGs under *S. fuliginea*. The significant *P* value of each KEGG term between the control and *S. fuliginea* infection samples was shown in the right heatmap. **b** KEGG enrichment analysis of the DAMs under *S. fuliginea*. The significant *P* value of each KEGG term between the control and *S. fuliginea* infection samples was shown in the left heatmap. All the KEGG terms were grouped into 11 metabolism-related categories, which were indicated by different color bars

Basing on their KEGG annotations, most of DEGs and DAMs were involved in 55 KEGG terms referring to 11 major metabolic categories. Significance value of each KEGG term in different comparisons was showed by heatmap. Under the *S. fuliginea* infection, most significantly enriched DEGs and DAMs were belonging to one amino acid-related pathway, four flavonoid-related pathways, two hormone-related pathways, three lipid-related pathways, one phenylpropanoid-related pathway, one pigments and vitamins-related pathway, two saccharide-related pathways, one terpenoid-related pathway, and one ubiquinone-related pathway (Fig. 3).

There are obvious differences in metabolic responses to *S. fuliginea* infection between BK2 and H136. For the flavonoid-related pathways, the genes belonging to ‘flavonoid biosynthesis’, ‘anthocyanin biosynthesis’, and ‘flavone and flavonol biosynthesis’ were significantly changed in BK2 and the genes belonging to ‘Isoflavonoid biosynthesis’ were significantly changed in H136. For the lipid-related pathways, the genes belonging to ‘arachidonic acid metabolism’, ‘linoleic acid metabolism’, and ‘α-linolenic acid metabolism’ were only significantly changed in H136. For the terpenoid-related pathways, the genes belonging to ‘terpenoid backbone biosynthesis’ were significantly changed in BK2 and genes belonging to ‘diterpenoid biosynthesis’ were significantly changed in H136 (Fig. 3a).

Then, we analyzed the DAMs respossive to *S. fuliginea* infection. For the flavonoid-related pathways, the DAMs belonging to ‘Isoflavonoid biosynthesis’ were significantly changed in BK2 and the genes belonging to ‘flavonoid biosynthesis’ and ‘flavone and flavonol biosynthesis’ were significantly changed in H136. For the lipid-related pathways, the DAMs belonging to ‘arachidonic acid metabolism’ and ‘α-linolenic acid metabolism’ were only significantly changed in H136. For the terpenpoid-related pathways, the DAMs belonging to ‘diterpenoid biosynthesis’ were significantly changed in BK2 (Fig. 3b).

### Variations in flavonoid metabolism under *S. fuliginea* infection

**Fig. 4:**
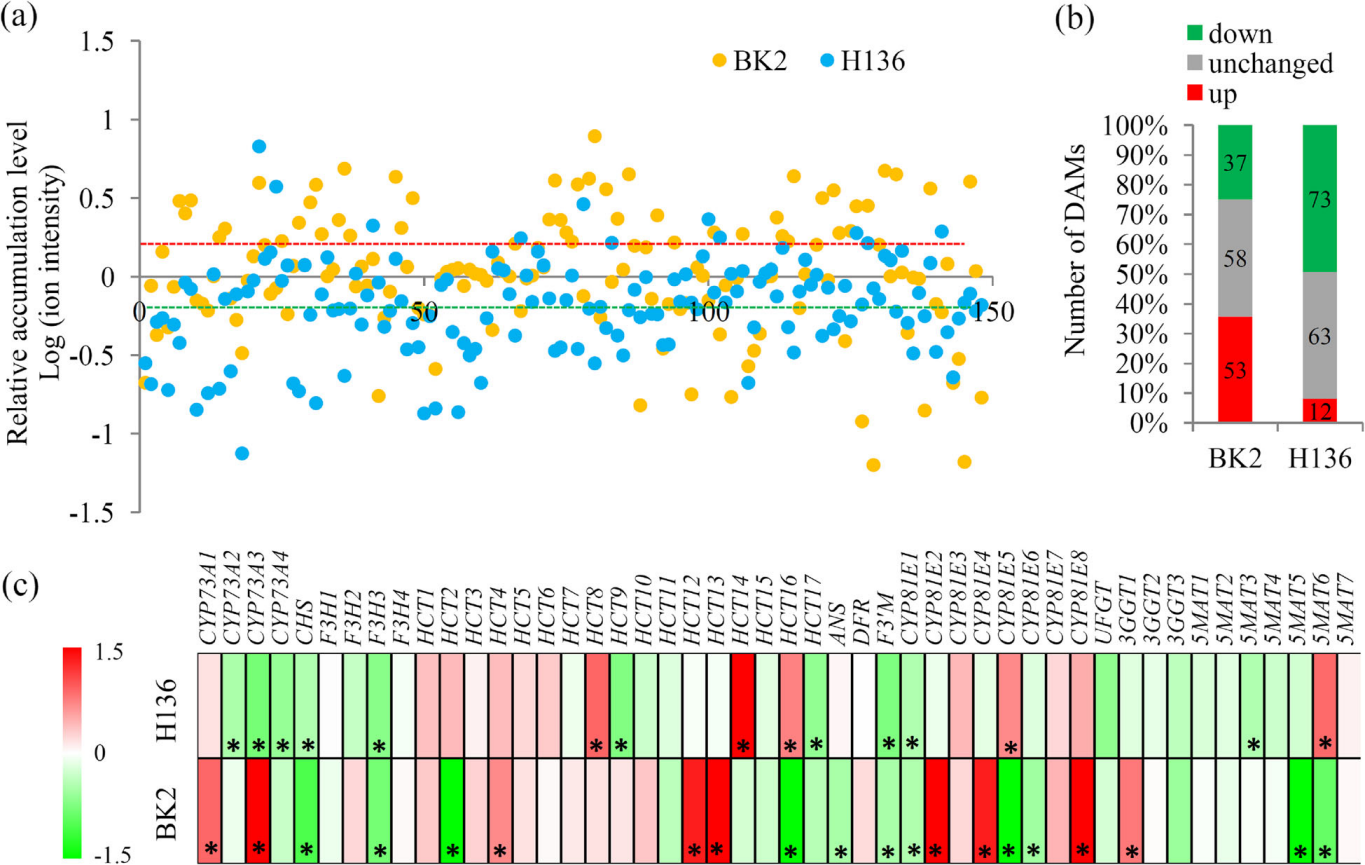
Integrated metabolomic and transcriptomic and transcriptomic analysis of the flavonoid biosynthesis. **a** The relative abundances of the metabolites belonging to flavonoid-related category. **b** The number of up- and down-regulated flavonoid-related metabolites under the *S. fuliginea* infection. **c** Differential expression of the key genes involved the flavonoid biosynthesis pathway. “*” represents significant differences (*P* < 0.05). The heatmap scale ranges from − 1.5 to + 1.5 on a *log*_*2*_ scale

In total, 148 flavonoid metabolism-related metabolites were identified by the metabolomic analysis (Fig. 4a). A majority of flavonoid metabolism-related metabolites, including 53 up- and 37 down-regulated metabolites, were significantly changed in BK2 under *S. fuliginea* infection. In H136, 85 flavonoid metabolism-related metabolites, including 12 up- and 73 down-regulated metabolites, were significantly changed under *S. fuliginea* infection (Fig. 4b).

Based on the transcriptomes, 48 genes encoding 11 flavonoid metabolism-related enzymes were identified in cucumber (Additional file 7). In total, 20 genes and 15 genes were significantly changed in BK2 and H136, respectively **(**Fig. 4c). In BK2, the number of up-regulated genes is similar to the down-regulated genes, which is consistent with corresponding DAMs. In H136, the number of down-regulated genes is larger than the up-regulated genes, leading to the low accumulation of flavonoid-related metabolites under *S. fuliginea* infection.

### Variations in hormone-related metabolisms under *S. fuliginea* infection

**Fig. 5:**
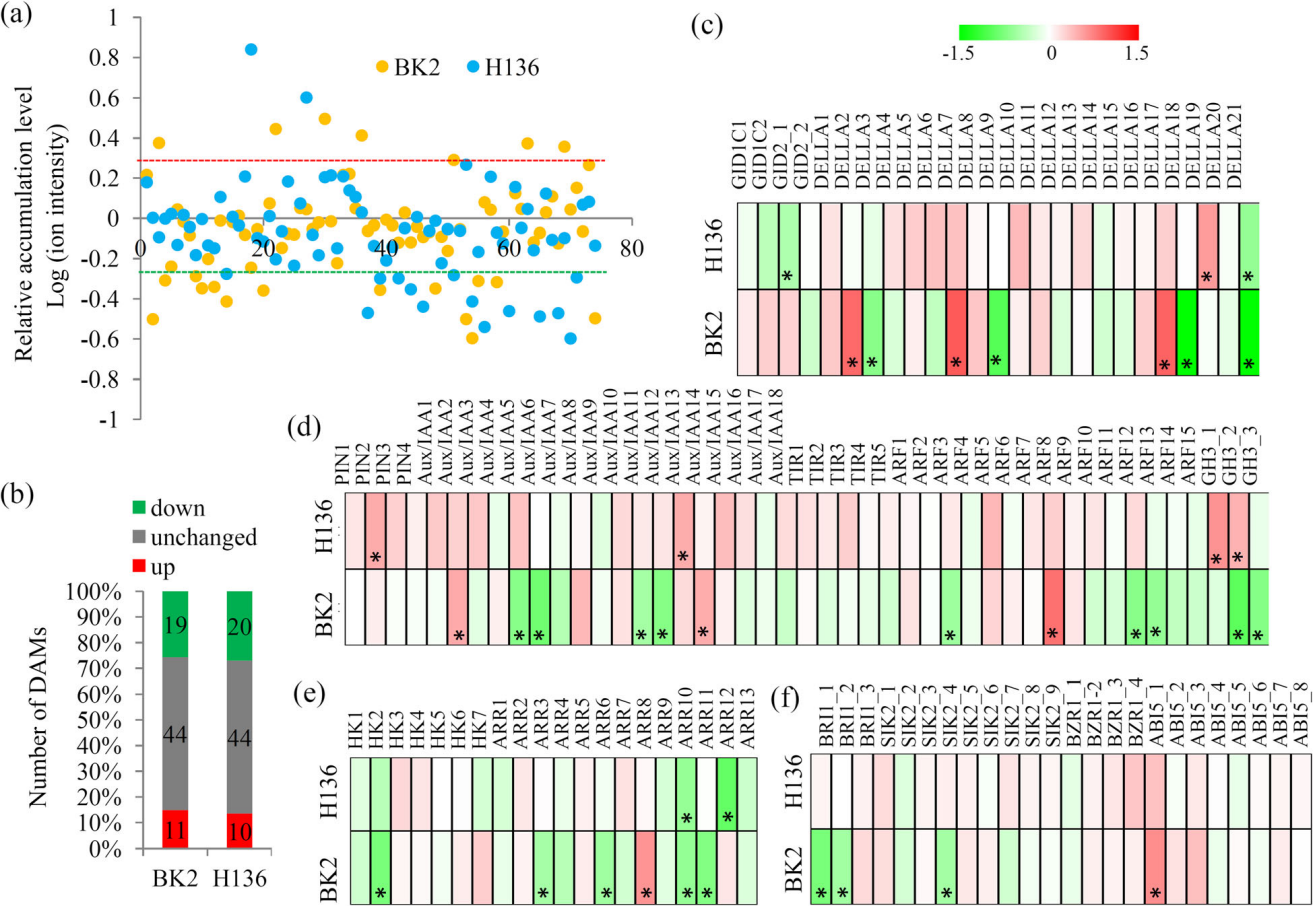
Integrated metabolomic and transcriptomic and transcriptomic analysis of the hormone-related pathways. **a** The relative abundances of the metabolites belonging to hormone-related category. **b** The number of up- and down-regulated hormone-related metabolites under the *S. fuliginea* infection. **c** Differential expression of the key genes involved various hormone signaling pathways. “*” represents significant differences (*P* < 0.05). The heatmap scale ranges from − 1.5 to + 1.5 on a log_2_ scale

Many hormone metabolism-related metabolites were identified by the metabolomic analysis (Fig. 5a). A majority of hormone metabolism-related metabolites were unchanged in both of BK2 and H136 under *S. fuliginea* infection (Fig. 5b).

In total, 25 genes encoding three important protein families involved in the GA signaling pathway, 45 genes encoding five protein families asscoicated with the auxin signaling pathway, 20 genes encoding two key emzymes involved in the cytokinin signaling pathway, 24 genes encoding four key components of the BR signaling pathway were identified by the transcriptomic analyses (Additional file 8). The detailed expression patterns of these hormone metabolism-related genes are shown in Fig. 5c-f.

### Variations in fatty acid metabolisms under *S. fuliginea* infection

**Fig. 6:**
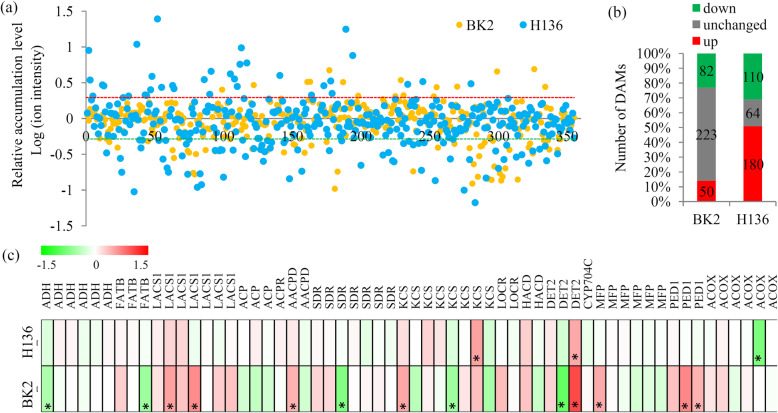
Integrated metabolomic and transcriptomic and transcriptomic analysis of the lipid metabolism. **a** The relative abundances of the metabolites belonging to hormone-related category. **b** The number of up- and down-regulated hormone-related metabolites under the *S. fuliginea* infection. **c** Differential expression of the key genes involved in various hormone signaling pathways. “*” represents significant differences (*P <* 0.05). The heatmap scale ranges from − 1.5 to + 1.5 on a log_2_ scale

A number of fatty acid metabolism-related metabolites were identified by the metabolomicanalysis (Fig. 6a). In BK2, only 132 fatty acid metabolism-related metabolites, including 50 up- and 32 down-regulated metabolites, were significantly changed under *S. fuliginea* infection. In H136, 290 fatty acid metabolism-related metabolites, including 180 up- and 110 down-regulated metabolites, were significantly changed under *S. fuliginea* infection (Fig. 6b).

In total, 60 genes encoding 15 key enzymes in the fatty acid metabolism pathway were identified by the transcriptomic analyses (Additional file 9). Expression pattern analysis showed that most fatty acid metabolism-related DEGs were identified in BK2 ranther than in H136 (Fig. 6c).

### Variations in diterpenoid metabolisms under the *S. fuliginea* infection

**Fig. 7:**
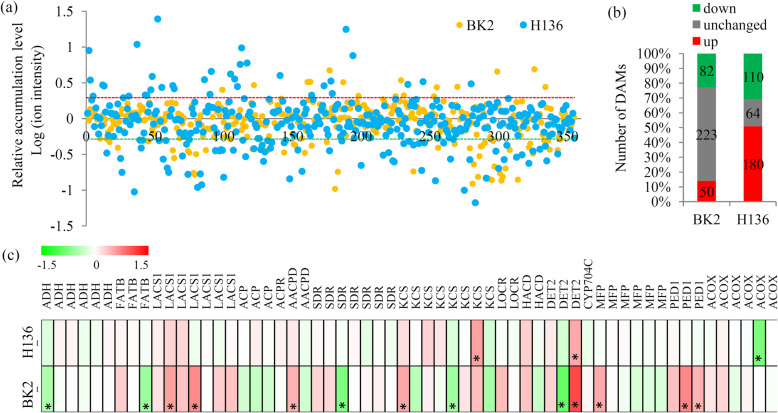
Integrated metabolomic and transcriptomic and transcriptomic analysis of the terpenoid biosynthesis. **a** The relative abundances of the metabolites belonging to terpenoid-related category. **b** The number of up- and down-regulated terpenoid-related metabolites under the *S. fuliginea* infection. **c** Differential expression of the key genes involved in terpenoid biosynthesis pathway. “*” represents significant differences (*P <* 0.05). The heatmap scale ranges from − 1.5 to + 1.5 on a log_2_ scale

A total of 183 diterpenoid metaboilism-related metabolites were detected in our study (Fig. 7a). In BK2, 59 diterpenoid metaboilism-related metabolites, including 20 up- and 39 down-regulated metabolites, were identified as DAMs. In H136, 86 diterpenoid metaboilism-related metabolites, including 19 up- and 67 down-regulated metabolites, were identified as DAMs (Fig. 7b).

Then, 20 genes encoding six diterpenoid metaboilism-related enzymes were identified by the transcriptomic analyses (Additional file 10). Interestingly, most diterpenoid metaboilism-related genes, including 5 up-regulated and 9 down-regulated genes, were siginificantly changed the *S. fuliginea* infection in BK2 (14 genes), and a small number of iterpenoid metaboilism-related genes, including 3 up-regulated and 3 down-regulated genes, were siginificantly changed by *S. fuliginea* infection in H136 (6 genes) (Fig. 7c).

### Identification of differentially expressed TFs in BK2 and H136 under *S. fuliginea* infection

A large number of TFs were reported to be involved in pathogen resistance [30]. In our study, 411 putative TF encoding genes belonging to eight major TF families were identified in cucumber. In BK2, 13 ERFs, 21 WRKYs, 11 bHLHs, 3 ARFs, 14 GATAs, and 18 NACs were identified as DEGs under *S. fuliginea* infection. In H136, 4 ERFs, 6 WRKYs, 8 bHLHs, 6 MYBs, 1 GATA were identified as DEGs under *S. fuliginea* infection (Additional file 11).

### Validation of gene expression and metabolite accumulation

To investigate the differences in expression levels of key genes, the relative levels of six randomly selected genes were determined by qRT-PCR analysis. The expression levels of the six genes were in agreement with the transcriptomic data (Additional file 12). The content of total flavonoids were determinedand and the contents of total flavonoids were shown in Additional file 13.

## Discussion

A number of cucumber genes and proteins responsive to *S. fuliginea* infection have been reported recently [14, 31]. Our previous study has mined several candidate genes associated with PM resistence in cucumber by specific length amplified fragment sequencing. The five candidate genes are F-box protein VBF, kinesin-4, calcium transporting ATPase 9, ras-related protein RABF1, and lysine-specific histone. None of them are related to primary and secondary metabolisms and the metabolic responses to *S. fuliginea* infection are still unknown. In the present study, cucumber PM-resistant variety ‘BK2’ and PM-susceptible variety ‘H136’ were used to reveal the differential responses to *S. fuliginea* infection.

In the past years, integrated omic analysis provided insights into various aspects of cucumber development and growth. For examples, combined transcript and metabolite analysis identified the mechanism involved in spider mite induced volatile formation in cucumber seedlings [32]. Transcriptomic and metabolomic analyses of cucumber fruits revealed an increasing in terpenoid glycosides during *Phytophthora capsici* infection [33]. A recent integrated metabolomic and transcriptomic analysis investgated the role of grafting with different rootstocks in fruit flavor of cucumber fruit [34]. With the advances in technology, more and more metabolites and genes could be detected by integrated omic analysis.

Screening of disease-resistant or -susceptible varieties is a high effective and environmental friendly approach to improve the disease resistance of crops. Transcriptome comparison of resistant and susceptible wheat identified several cell wall- and flavonoid biosynthesis-related genes responsive to PM infection [35]. To screen the genes related to downy mildew (DM), one DM-resistant cultivar D9320 and one DM-susceptible cultivar D0401 were used, identifying a cucumber DM resistance-related gene *CsERF004* [36]. Taking advantage of cucumber aphid resistance cultivar EP6392, several flavonoid biosynthesis-, amino acid metabolism- and sugar metabolism-related genes were reported to be associated with aphid resistance [37]. Expression profile analysis of a cucumber watermelon mosaic virus susceptible line ‘Europe 8’ identified a leaf-specific expressed transcription factor CsTCP14 that played a key role in response to foliage disease [38]. For PM disease, several PM resistant materials have been applied in cucumber production. For examples, three cucunber inbred lines, S1003, S1001, and S05, were applied to map the major locus for PM resistance, identifying a functional plant-specific integral membrane protein gene *CsMLO1* [28]. In our study, a resistant cultivar BK2 and a susceptible cultivar H136 were used to screen DEGs and DAMs under *S. fuliginea* infection [13]. In the H136C vs BK2C comparison, 3029 DEGs and 255 DAMs were identified, suggesting a basic distinct between these two cultivars before *S. fuliginea* infection. In the H136T vs BK2T comparison, 5832 DEGs and 971 DAMs were identified, providing a large number of candidate genes and metabolites associated with PM resistance.

A number of primary and secondary metabolites have been reported to be involved in virus resistance in various crops. In melon, the content of phenolics, flavonoid and tannins, as well as their synthesis-related genes, were largely induced by *Podosphaera xanthii* infection [39]. In plants, flavonoids play important roles in response to various environmental stresses [40]. A biostimulant prepared from *Ascophyllum nodosum* extract suppresses PM of strawberry by increasing the total phenolic and flavonoid contents [41]. Further study pointed out that exogenous application of pesticide containing flavonoids inhibited the spore germination of PM in *Hordeum vulgare* [42]. A previous study has showed that the expression of several flavonoid metabolism-related genes significantly changed under PM infection in wheat [35]. In our study, many flavonoid biosynthesis-related metabolites and genes were dectected in cucumber plants. Intreastingly, there were great differences in accumulation of flavonoid biosynthesis-related metabolites between BK2 and H136 (Fig. 4b). Increasing in *S. fuliginea* infection-induced flavonoid contents might play an esstential role in formation of resistant quality of BK2. In Tibetan hulless barley, gene co-expression combined with metabonomic analysis reveals the resistance responses to PM infection [43]. Expression of the flavonoid pathway-related genes, such as *4CL* and *CHS*, was considered to be associated with suppression of induced resistance in cucumber [18]. In our study, the *CHS* gene was identified by the transcriptomic analysis, and its expression was down-regulated in both of BK2 and H136. In wheat, flavonoid 3-hydroxylase (*F3H*), converting flavonone to dihydroflavonol, was up-regulated under PM infection [35]. In cucumber, *F3H3* was significantly down-regulated in both of BK2 and H136, suggesting difference between monocotyledonous and dicotyledonous plants. Several important flavonoid pathway genes showed significant differential expression between BK2 and H136, provided a probable explaination for the differential accumulation of flavonoid-related metabolites.

Various phytohormones have been reported to be involved in the responses to pathogen stresses [44]. For examples, the level of salicylic acid, a broad-spectrum antipathogen defense-related phytohormone, was significantly elevated in PM *Cucurbita pepo* plants [45]. In our study, a number of hormone-related metabolites and genes were identified in cucumber. Compared to metabolite accumulation, expression of hormone-related genes were significantly changed by *S. fuliginea* infection. Interestingly, more hormore-related DEGs were detected in BK2 ranther than H136, suggesting a violent response of horomne signaling pathways to *S. fuliginea* infection in PM-resistant variety. Auxin signaling is finely manipulated during pathogen infections, and several auxin signaling-related factors, such as F-box receptors and auxin response factors (ARFs), were responsive to PM infection [46, 47]. Our data suggested that ARF-mediated auxin signaling pathway was involved in the PM-resistance of cucumber plants. Abscisic acid is another important hormone that negatively regulates post-penetration resistance of model plant *Arabidopsis* to the PM fungus [48]. BR insensitive 1 (BRI1) is the key BR receptor and plays critical roles in BR signaling [49]. In our study, two BRI1 encoding genes were significantly reduced by *S. fuliginea* infection in BK2, suggesting a inhibition of BR signaling in the PM-resistant variety.

Previous studies showed that fatty acid metabolism as well as lipid signaling have a close relevance to PM resistance [50]. For example, several wheat fatty acids, such as C12:0, C18:1, C18:2 and C20:2, were significantly changed by PM infection [51]. Further study showed that the regulation of fatty acid metabolism was requried for glycerol-induced PM resistance in wheat [50]. In our study, the number of fatty acid metabolism-related DAMs in H136 (290 metablites) was larger than that in BK2 (132 metablites), indicating an active fatty acid metabolism in the PM-susceptible variety. In barley and *Arabidopsis*, expression of 3-KETOACYL-CoA SYNTHASES (KCS) encoding gene provides wax signals for germination of PM fungi spores [52]. In cucumber, eight differentially expressed *KCS* genes were identified, suggesting that wax signals might also play an important role in response to *S. fuliginea* infection. Two long chain acyl-CoA synthetase (LACS) encoding genes were siginificantly up-regualated in BK2, indicating that long chain fatty acids might be involved in the response of cucumber plants to pathogens [53]. Under microbial infection, diterpenoids was reproted to be required for a phenomenon known as systemic acquired resistance (SAR) [54]. In cucumber, most of diterpenoid biosytnhesis-related genes were significantly changed in BK2, suggesting a difference in SAR between BK2 and H136.

Several TFs were reported to be involved in PM resistence in various plants. For examples, MED25 and JAZ1 from wheat, WRKY52 from *Arabidopsis*, ERF1-V in *Harnaldia villosa*, TIFY9 and NAC042 from *Vitis vinfera*, were responsive to PM resistance [31, 55–60]. In our study, a large number of differentially expressed TFs were identified in the two cucumber varieties, providing some potential TFs for the improvement of PM resistance.

## Conclusions

Comparative metabolomic and transcriptomic analyses revealed the variations in primary and secondary metabolisms of cucumber plants under *S. fuliginea* infection. A large number of DAMs and DEGs associated with flavonoid, hormone, fatty acid and diterpenoid metabolisms were identified between BK2 and H136. Our data suggested that four major types, including flavonoids, hormones, fatty acids and diterpenoid, could be developed to control the PM disease. Furthermore, several differentially expressed TFs were also identified, providing novel candidate regulators of PM resistance. Our study may aid in better understanding of the differences between cucumber PM-resistant variety ‘BK2’ and PM-susceptible variety ‘H136’, and promoting the breeding of PM resistant varieties.

## Material & Methods

### Plant material and sampling

Cucumber PM-resistant variety ‘BK2’ and PM-susceptible variety ‘H136’ were used for our study. The detail information the two varieties were described by our previous paper [13]. Cucumber plants were grown in a growth chamber in Zhejiang Academy of Agriculture Sciences. The leaves at the three-leaf stage were selected and sprayed with *S. fuliginea* solution for inoculation. The treatment solution was prepared with 2 × 10^6^ sporangia/mL *S. fuliginea*, 5 mM glucose, and 2.5 mM KH_2_PO_4_. The sterile water was used as control solution. The seedlings inoculated with *S. fuliginea* solution and control solution were put in a dark room for 24 h and separated with plastic film. Ten independent seedlings per replicate were harvested at 48 h post inoculation and were immediately frozen in liquid N_2_ until use.

Four different sample groups, including BK2 treated with *S. fuliginea* solution (BK2T), BK2 treated with control solution (BK2C), H136 treated with *S. fuliginea* solution (H136T), H136 treated with control solution (H136C), were used. For transcriptome sequencing, four library groups (three libraries for each group) were constructed for RNA sequencing. For metabolome analysis, four sample groups (ten samples for each group) were prepared for metabolite extraction.

### RNA sequencing and expression analysis

Total RNA extraction was performed according to the previous publication [61]. Transcriptome sequencing was performed on Illumina HiSeqTM 4000 platform in LC-Bio Co. (Hangzhou, China) according to the protocol. For quality checking, various parameters, such as Q20, Q30, N50 and GC content, were evaluated to verify the reads in FastQC format. Expression level of each transcript was calculated using the reads per kilo bases per million reads (RPKM) method. The genes from each sample group were annotated by searching the cucumber genome database in NCBI (ID: ASM407v2). The baseMean value was used to calculate the sequencing depth of each transcript normalized to the library size. Genes with significant expression changes larger than twofold between two BK2 and H136 at different treatments were considered as DEGs by DESeq2 v.1.6.3 [62].

### Enrichment analysis and K-means clustering of the DEGs

Enrichment analysis of DEGs were performed with Gene Ontology (GO, http://www.geneontology.org/) and Kyoto Encyclopedia of Genes and Genomes (KEGG, https://www.kegg.jp/) databases. GO enrichment analysis of the DEGs was performed using GOseq R package with Wallenius non-central hyper-geometric distribution. The KEGG enrichment analysis of DEGs were performed using KOBAS software. Two-tailed Fisher’s exact method was applied to analyze the functional enrichments of GO and KEGG terms. GO and KEGG categories with a corrected *P* value lower than 0.05 was considered as a significant term.

The number of clusters was calculated by ClusGap R function-cluster package (v.2.0.5). Then, K-means clustering program was applied to check clusters basing on relative gene expression data. The results of clustering were performed using MeV program (V4.9.0).

### Metabolite extraction and quality control (QC) sample preparation

The leave samples from different groups (25 mg each, *N* = 10), were put into a micro tube and added with 800 μL of pre-colded methanol (50%). The mixed sample solution was shaken with 1800 shakes per min for 60 s with a 2010 SPEX Geno/Grinder (SamplePrep, Metuchen, USA). Then, the sample solution was added with 500 μL of pre-colded chloroform/methanol/water (v:v:v, 1:3:1), shaken in dark for 10 min on ice, and processed to ultrasonication for 5 min at 4 °C. The supernatant was obtained, vacuum-dried, and resuspended in 50% methanol. A QC sample was obtained by mixing an equal volume of each experimental sample.

### UPLC-MS/MS analysis of the extractions

An UHPLC 1290 Agilent system (Santa Clara, CA, USA) and a high-resolution MS equipped with an ESI interface (Q Exactive Orbitrap, Santa Clara, CA, USA) were integrated for UPLC-MS/MS analysis. An UPLC T3 column (ACQUITY, 100 mm × 2.1 mm, 1.8 μm, Waters, UK) was used for reversed phase separation. The parameters of reversed-phase separation and gradient elution were set according to the previous publication [63].

A high-resolution MS/MS SCIEX Triple-TOF-5600 plus system was applied to identify metabolites eluted from the reversed phase column. The other analytic parameters were set according to the previous publication [63]. A QC sample was uploade to the system after every 10 experimental samples to test the stability of MS/MS system.

### Untargeted metabolomic analysis

MS/MS data analyses, including peak picking, peak grouping, retention time (RT) adjusting, second peak grouping, isotopes annotation, and adducts annotation, were carried out using SCMS software. Then, the LC-MS/MS raw data were converted into mzXML format by metaX software implemented with R toolbox [64]. Each detectable ion was recognized by integrated RT and *m/z* data, generating the intensity of each peak. For metabolite annotation, the exact *m/z* of each metabolite was matched to the data from the criterion: a mass difference between the detected metabolites and the database value was less than 10 ppm. The molecular formulas of metabolites were further validated by the isotopic distribution measurements and a in-house fragment spectrum library.

### Real-time PCR and flavonoid content

Real-time PCR validation was carried out according to the standard process on a DNA Sequence Detection System (ABI PRIM 7700). In briefly, independent RNAs from the same four sample groups were used for real-time PCR analysis. A cucumber ACTIN sequence was used as the internal standard to calculate relative fold differences by the values of comparative cycle threshold (2^−ΔΔCt^). The expression analysis was performed for three biological replications. The primer information has been listed as Additional file 14.

The total flavonoid contents were measured using HPLC assay. In briefly, cucumber samples were extracted with 75% ethanol at 37 °C for 30 min. Cell debris was discarded by centrifugation, and the supernatant was used for HPLC analysis. For HPLC analysis, the supernatant was uploaded to a Waters HPLC e2695 series (Waters, USA). HPLC was performed on an XBridge C18 (4.6 mm × 250 mm) column. The elution was performed using solution A [H_2_O: TFA (1000: 1)] and B [C2H3N: CH3OH (9: 1)]. Rutin were purchased from Sigma-Aldrich (St. Louis, MO, U.S.A.) [65].

### Statistical analysis of the DAMs

Student *t*-test was performed to evaluate the differences in the metabolite levels between two sample groups. An FDR (Benjamini–Hochberg) method was used to adjust the *P* value for multiple tests. The detailed method of statistical analysis was performed according to the previous publication [64].

## Supplementary Information

**Additional file 1: Table S1** The detailed information of RNA sequencing.

**Additional file 2: Table S2** The detailed information of the mapped reads from different samples.

**Additional file 3: Table S3** The detailed information of 24,317 cucumber genes with annotation.

**Additional file 4: Table S4** The detailed information of all identified metabolites.

**Additional file 5: Figure S1** Analysis of several quality parameters of the metabolomes.

**Additional file 6: Table S5** The detailed information of metabolites with KEGG annotation.

**Additional file 7: Table S6** The detailed information of flavonoid metabolism-related genes.

**Additional file 8: Table S7** The detailed information of hormone-related genes.

**Additional file 9: Table S8** The detailed information of fatty acid metabolism-related genes.

**Additional file 10: Table S9** The detailed information of diterpenoid metabolism-related genes.

**Additional file 11: Table S10** The number of TFs.

**Additional file 12: Figure S2** Expression validation of the key genes involved in responses to *Sphaerotheca fuliginea* infection.

**Additional file 13: Figure S3** Differential accumulation of total flavonoids.

**Additional file 14: Table S11** The primer information.

## Data Availability

The raw sequence data has been submitted to the NCBI Short Read Archive with accession numbers SRP212890.

## Abbreviations

ABA: Abscisic acid
ARF: Auxin response factor
BR: Brassinolide
BRI1: BR insensitive 1
CTK: Cytokinin
CV: Coefficent of variation
DAM: Differentially accumulated metabolite
DEG: Differentially expressed gene
DM: Downy mildew
GA: Gibberellin
GO: Gene Ontology
KCS: 3-KETOACYL-CoA SYNTHASES
KEGG: Kyoto Encyclopedia of Genes and Genomes
LACS: Long chain acyl-CoA synthetase
PC: Principal component
PW: Powdery mildew
TIC: Total ion chromatogram
SAR: Systemic acquired resistance
RPKM: Reads per kilo bases per million read
RT: Retention time
QC: Quality control
