# Correction to: Comparative analysis of powdery mildew resistant and susceptible cultivated cucumber (*Cucumis sativus* L.) varieties to reveal the metabolic responses to *Sphaerotheca fuliginea* infection

**DOI:** 10.1186/s12870-021-02873-2

**Published:** 2021-02-11

**Authors:** Peng Zhang, Yuqiang Zhu, Shengjun Zhou

**Affiliations:** Institute of Vegetable, Zhejiang Academy of Agriculture Sciences, Hangzhou, China

**Correction to: BMC Plant Biol (2021) 21: 24**

**https://doi.org/10.1186/s12870-020-02797-3**

Following publication of the original article [1], the authors identified an error. Figures 6 and 7 are the same. The correct figure 7 is given below.

The original article [1] has been corrected.

**Correct Figure 7:**


