# Supplementary material for: Comparative analysis of powdery mildew resistant and susceptible cultivated cucumber (Cucumis sativus L.) varieties to reveal the metabolic responses to Sphaerotheca fuliginea infection
Source: BMC Plant Biol. 2021 Jan 7;21:24. doi: 10.1186/s12870-020-02797-3 (PMC7791650; doi:10.1186/s12870-020-02797-3)
Supplement: Supplementary file 5 — Additional file 5: Figure S1 Analysis of several quality parameters of the metabolomes. [file 12870_2020_2797_MOESM5_ESM.pdf]

Comparative analysis of powdery mildew resistant and susceptible cultivated cucumber (*Cucumis sativus* L.) varieties to reveal the metabolic responses to *Sphaerotheca fuliginea* infection

Peng Zhang, Yuqiang Zhu, Shengjun Zhou*

[1] Institute of Vegetable, Zhejiang Academy of Agriculture Sciences, Hangzhou, China

* Corresponding author:

Shengjun Zhou

Email Address: yinxiang0586@sohu.com

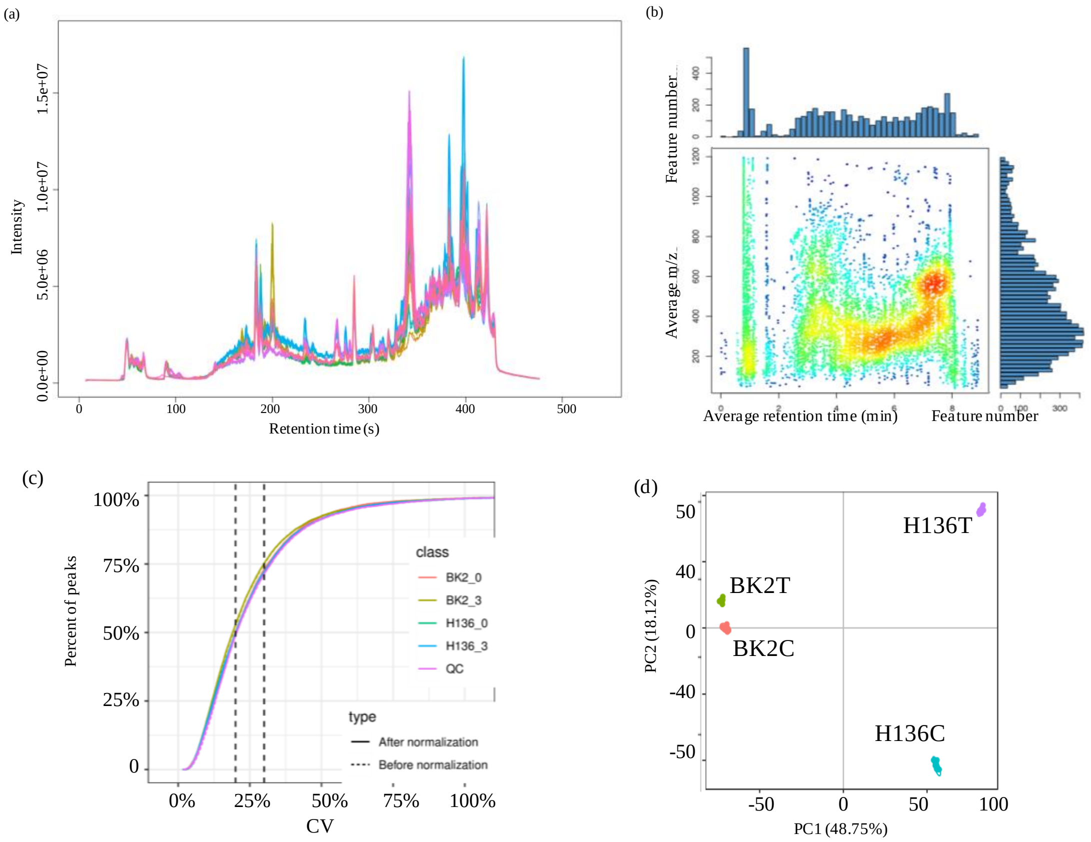

Figure S1 Analysis of several quality parameters of the metabolomes. (a) The total ion chromatograms of all the samples. (b) *m/z* widths and retention-time widths for the metabolomes. (c) CV distribution of the data from different samples were shown.
